# Wide-Area Debris Field and Seabed Characterization of a Deep Ocean Dump Site Surveyed by Autonomous Underwater Vehicles

**DOI:** 10.1021/acs.est.3c01256

**Published:** 2023-06-15

**Authors:** Sophia T. Merrifield, Sean Celona, Ryan A. McCarthy, Andrew Pietruszka, Heidi Batchelor, Robert Hess, Andrew Nager, Raymond Young, Kurt Sadorf, Lisa A. Levin, David L. Valentine, James E. Conrad, Eric J. Terrill

**Affiliations:** † Scripps Institution of Oceanography, La Jolla, California 92037, United States; ‡ University of California, Santa Barbara, Santa Barbara, California 93106, United States; ¶ U.S. Geological Survey, Pacific Coastal and Marine Science Center, Santa Cruz, California 95060, United States

**Keywords:** ocean dumping, marine robotics, side-scan sonar, dichlorodiphenyltrichloroethane (DDT), marine debris

## Abstract

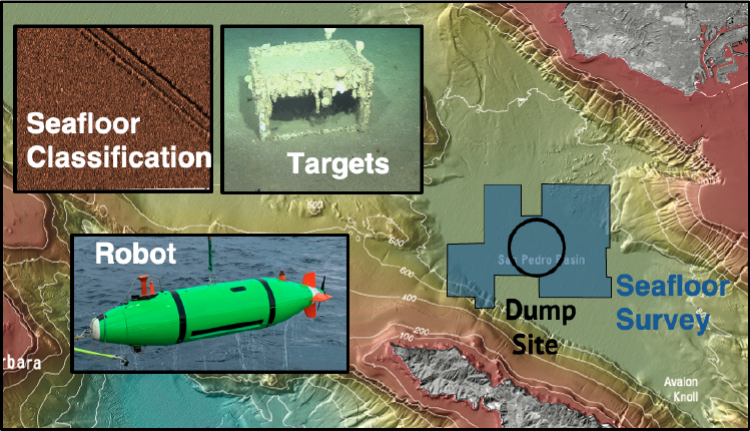

Disposal of industrial and hazardous waste in the ocean was a pervasive global practice in the 20th century. Uncertainty in the quantity, location, and contents of dumped materials underscores ongoing risks to marine ecosystems and human health. This study presents an analysis of a wide-area side-scan sonar survey conducted with autonomous underwater vehicles (AUVs) at a dump site in the San Pedro Basin, California. Previous camera surveys located 60 barrels and other debris. Sediment analysis in the region showed varying concentrations of the insecticidal chemical dichlorodiphenyltrichloroethane (DDT), of which an estimated 350–700 t were discarded in the San Pedro Basin between 1947 and 1961. A lack of primary historical documents specifying DDT acid waste disposal methods has contributed to the ambiguity surrounding whether dumping occurred via bulk discharge or containerized units. Barrels and debris observed during previous surveys were used for ground truth classification algorithms based on size and acoustic intensity characteristics. Image and signal processing techniques identified over 74,000 debris targets within the survey region. Statistical, spectral, and machine learning methods characterize seabed variability and classify bottom-type. These analytical techniques combined with AUV capabilities provide a framework for efficient mapping and characterization of uncharted deep-water disposal sites.

## Abstract 2

This study presents an analysis of a wide-area side-scan sonar survey conducted with autonomous underwater vehicles (AUVs) at a deep water dump site in the San Pedro Basin, California.

## 1 Introduction

The historical practice of ocean dumping in United States waters has been driven by both the economics of hazard disposal and a desire to place contaminants far from population centers. With the formation of the Environmental Protection Agency (EPA) in 1970 and a growing body of science that revealed the negative impacts of dumping to the environment, the process became federally regulated with strict guidelines and a permit approval process when the 1972 Marine Protection, Research, and Sanctuaries Act was passed by Congress (MPRSA^1^). Twenty-six years later, an outright ban of industrial waste dumping was enacted through the Ocean Dumping Ban Act of 1998 (Public Law 100-688), with remaining permitted dumping activities limited to sewage and dredge spoil disposal. Prior to legal protection, the ocean was a favored disposal site for industrial waste, with 23 known sites used offshore the Atlantic, Gulf, and Pacific Coasts.^2^ Disposal took the form of both containerized waste and bulk dumping.^3^

Southern California’s coastal ocean hosts productive fisheries, is home to now protected ecosystems, and has an established tourism industry that is based on coastal recreation. Dump sites offshore California were established as early as the 1930s, became regulated in 1961, and were used for a variety of industrial purposes including disposal of waste from oil and gas production and the chemical manufacturing industry. Concern over these historical practices and their impact on the environment were described to the California Regional Water Quality Board in 1985.^5^ The report documented extensive regulated dumping of a variety of bulk and containerized materials and the possibility of *short-dumping*, disposal prior to reaching the sanctioned dumping location. The San Pedro Basin, located in Southern California waters between Santa Catalina Island and Palos Verdes Peninsula at depths ranging from 600 to 900 m, was a dump site for military munitions^6^ and a range of industrial wastes, including waste from refineries and chemical production. This included waste byproduct containing the pesticide dichlorodiphenyltrichloroethane (DDT), generated by the Montrose Chemical Corporation. Between 1947 and 1961, up to 700 t of DDT contained within acid sludge were dumped.^5^ While only accounting for a small fraction of the total overall waste recorded as dumped, the DDT waste byproducts are of particular concern due to the long life of the chemical. DDT is now well understood to be both toxic and stable with long-lasting negative environmental impacts^7^ including contamination of food webs, altering reproduction cycles, and contributing to cancer within wildlife.^8,9^ As an endocrine disrupter and immune suppressor, recent studies have demonstrated human health linkages between DDT exposure from fish consumption and breast cancer in women that can be passed down through generations.^10^ Interest is growing to develop a long-term strategy to assess the risk this dump site poses for both the surrounding marine ecosystem^11^ and the coastal population of Southern California. To date, no systematic survey of the locations and conditions of the dump site has been conducted, due in part to the historical technical challenges associated with deep water survey. Although the presence of DDT in seafloor sediment samples has been recognized for decades,^12,13^ it was only recently that surveys investigated whether barrels found on the seafloor could be a source.^14^ Several studies have been published documenting the negative impacts of DDT to the marine food web in Southern California including birds,^15^ dolphins,^9^ and humans.^16^

Below 800 m depth, the San Pedro Basin contains nearly anoxic waters^17,18^ bound by steep sidewalls to the northeast and southwest. The basin floor is generally smooth and flat, except for a hummocky area^19^ in the northeast region comprised of bedrock blocks and other debris from the Palos Verdes debris avalanche.^20^ Primary terrigenous sediment input to the basin is from the Redondo and San Pedro Sea Valleys to the north; only minor amounts of sediment are derived from Santa Catalina Island and areas to the east, mainly by mass wasting of the slope areas. The combined Holocene terrigenous and hemipelagic sedimentation rate in the study area is about 40 cm/ka.^21^ Currents in the area are weakly connected to the surface winds and near the seabed are on average equatorward but also subject to both tidal fluctuations and remotely forced poleward flows on time scales of 20–30 d.^22−24^ Particle deposition from biogeochemical cycles results in mass fluxes of approximately 500 mg d^–1^ m^–2^, resulting in layers of fine sediment on the seafloor.^18^ The deep basin circulation was studied using chemical tracers, showing years of basin stagnation where exchange with outside waters occurred dominantly through eddy diffusion and years of basin flushing where advection was dominant in deep water properties.^25^ Although several oceanographic studies have been conducted in the region, it is only with the advent of new autonomous underwater vehicle observational techniques, data storage, and computer processing improvements that very high resolution seabed (scales of centimeters) characterization can be conducted over wide areas. An overarching challenge for future studies will be to use high-resolution survey information to characterize bottom type and mobility and relate it to basin circulation and exchange rates.

AUVs equipped with side-scan sonars have long duration and deep-water capabilities making them effective tools for wide-area surveys of deep ocean sites. Often used for archeological work^26^ in search of large objects such as aircraft or shipwrecks, side-scan sonars produce backscatter intensity maps of the seafloor which resemble optical images. As wide-area surveys expand over 100 s of square kilometers, a variety of techniques have been developed to mosaic neighboring imagery and interpret resulting larger-scale maps. These techniques include unsupervised methods,^27^ filtering and equalization,^28^ and statistical analysis.^29^ Object detection also has a rich literature which includes machine learning^30−35^ and deep learning.^36−38^ Recent target detection algorithms using supervised deep learning techniques^36^ explore several deep convolutional neural networks (CNNs) as feature extractors in combination with support vector machines (SVMs) to locate and identify different types of mines. Transfer learning approaches^38^ using a pretrained CNN model with the object detection algorithm You Only Look Once (YOLO) have shown skill in detecting and classifying targets in debris fields. While the applications of machine learning on side-scan sonar data are growing, few studies have looked at wide area surveys with distributed, small targets (less than a few meters) on ocean basin scales.

This paper documents analytical techniques for a wide-area survey where dumped debris targets are small and distributed. An example of the challenge of automated detection algorithms is shown in raw side-scan imagery (Figure 2) where background texture, small bright targets, and a large object are in close proximity. Our method equalizes imagery across wide areas, characterizes the background seabed using statistical, spectral, and machine learning techniques, and identifies objects using a classifier based on previous ROV imagery of ground truth targets. We interpret spatial patterns in the seabed and debris targets to inform further studies of chemical, biological, and transport impacts and for upcoming target imaging from robotic platforms to characterize the types and conditions of the dumped materials and their effects on biota.

## 2 Methods

**Figure 1:**
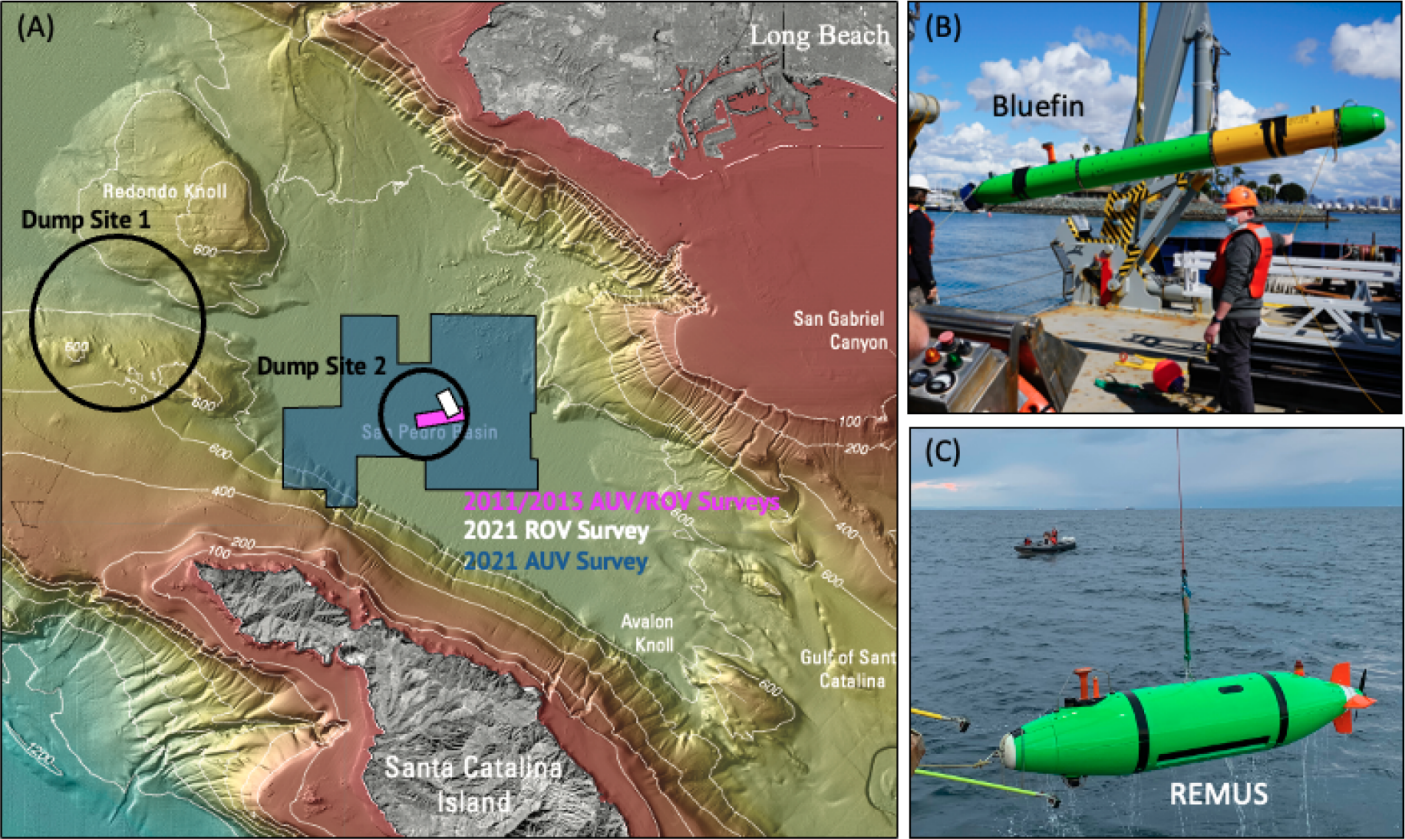
(A) Map of the ocean dump sites #1 and #2 (black), previous survey footprints from 2011 and 2013 (pink) and 2021 (white), and a 2021 survey footprint (dark blue) located in the San Pedro Basin, CA. The two vehicles that performed the wide-area survey are a (B) Bluefin 12D and a (C) REMUS 6000. Adapted with permission from ref (4). Copyright 2021 USGS.

In April 2021, the R/V Sally Ride and two deep-water AUVs completed a wide-area seafloor mapping study over the San Pedro Basin dump site *#*2 (Figure 1A). The AUVs operated in a lawn-mower survey pattern extending beyond the sanctioned dump site to map seafloor targets and characterize large-scale patterns in dumping practices. Wide-area surveys generate large data sets that benefit from automated routines for efficient and near real time target and classification detection and seabed characterization. A flowchart of the data processing algorithms developed for this survey is shown in Figure 3. More details of the vehicle surveys and data processing are given in the sections below and in the Supporting Information.

**Figure 2:**
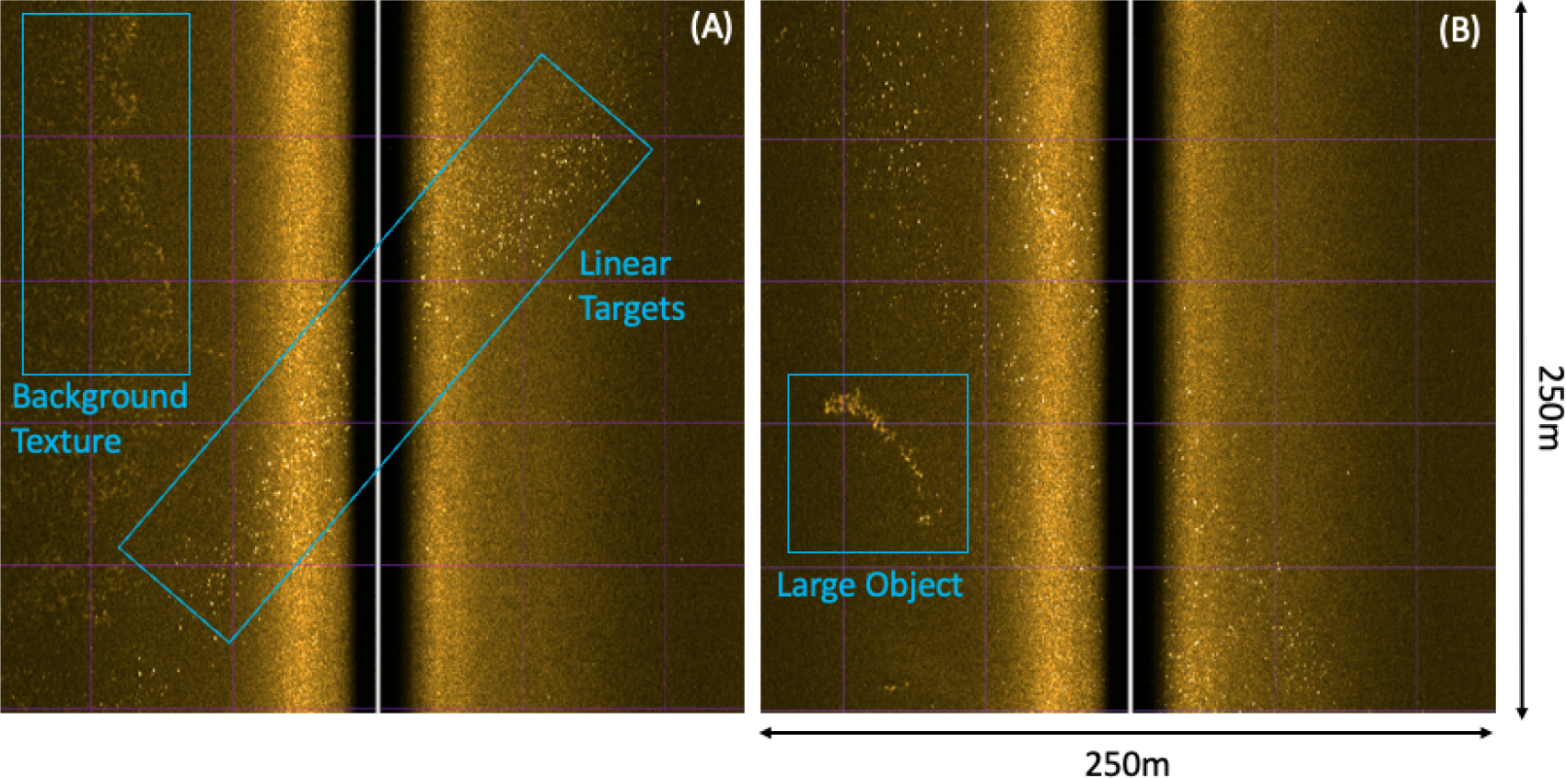
Raw side-scan imagery: (A) Examples of background textures and targets organized in a line and (B) a large object for visualization of the classification challenges of small distributed targets.

**Figure 3:**
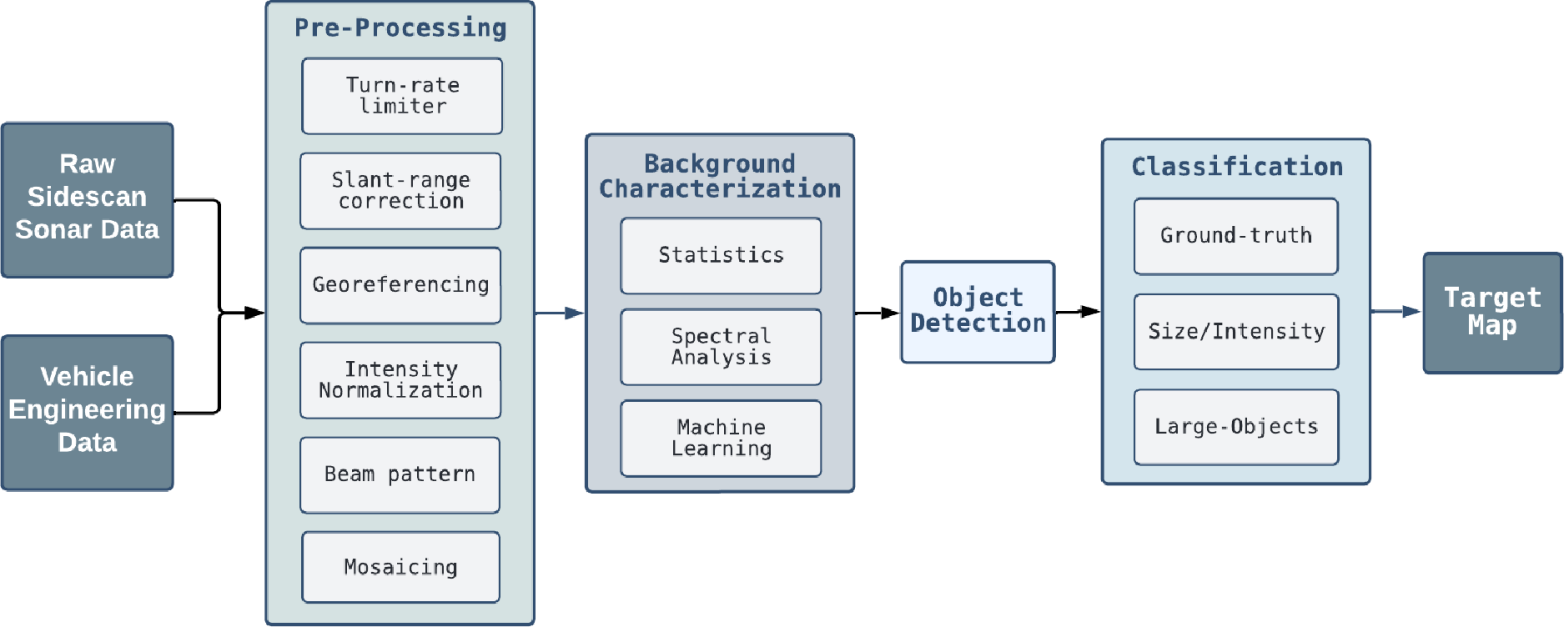
Algorithm flowchart depicting the processing chain from raw side-scan data to a target map.

### 2.1 Autonomous Underwater Vehicle (AUV) Surveys

Two AUVs equipped with side-scan sonars (EdgeTech Corporation, Wareham, MA) were used for the wide-area survey, a Bluefin 12D (General Dynamics Mission Systems, Fairfax, VA) (Figure 1B) and a REMUS 6000 (Huntington Ingalls Industries (HII), Pocasset, MA) (Figure 1C). Details of each vehicle’s side-scan sonar frequencies, ranges, and resulting pixel resolution are given in Table 1. The AUVs performed a total of 15 dives, surveying a 150-km^2^ region (Figure 1A). Within dives, individual files represent one trackline of a lawnmower sampling pattern designed for 200% coverage. Swath widths and row spacings were 150 m and 75/225 m for the Bluefin and 200 m and 100/300 m for the REMUS, respectively. The Bluefin side-scan ping rate was 5 Hz, and leg lengths were typically 1–3 km. The Bluefin surveyed at a speed of 2.2 m s^–1^ and an altitude of 15 m. The REMUS side-scan ping rate was 3 Hz, and leg lengths were similar in length to the Bluefin surveys. The REMUS surveyed at a fixed speed of 1.8 m s^–1^ and an altitude of 20 m.

**Table 1:** AUV Survey and Side-Scan Details

Vehicle	Speed	Altitude	Frequency	Range	Along-track res.	Cross-track res.
Bluefin 12D	2.2 m/s	15 m	410 kHz	150 m	45 cm	1.5 cm
Remus 6000	1.8 m/s	20 m	230 kHz	200 m	56 cm	1.9 cm

Both AUVs used inertial navigation and ship to vehicle Ultrashort Baseline (USBL) for position updates, a Kongsberg HIPAP system (27 kHz) and a Sonardyne Ranger System (30 kHz). The USBL was used to update vehicle position during descent and leading up to the first survey line until a stable flight with bottom lock from the Doppler Velocity Log (DVL) was acquired to support an internal inertial navigation system (INS). In-mission, USBL updates were made at the operator’s discretion if tracks from the surface USBL did not correlate with the INS position relayed by acoustic telemetry messages. Individual dives averaged 8–10 h in duration, and navigational offsets between dives were estimated using large targets as references and averaged 30 m. Further details on side-scan preprocessing are given in the Supporting Information.

## 3 Results and Discussion

### 3.1 Seabed Characterization

**Figure 4:**
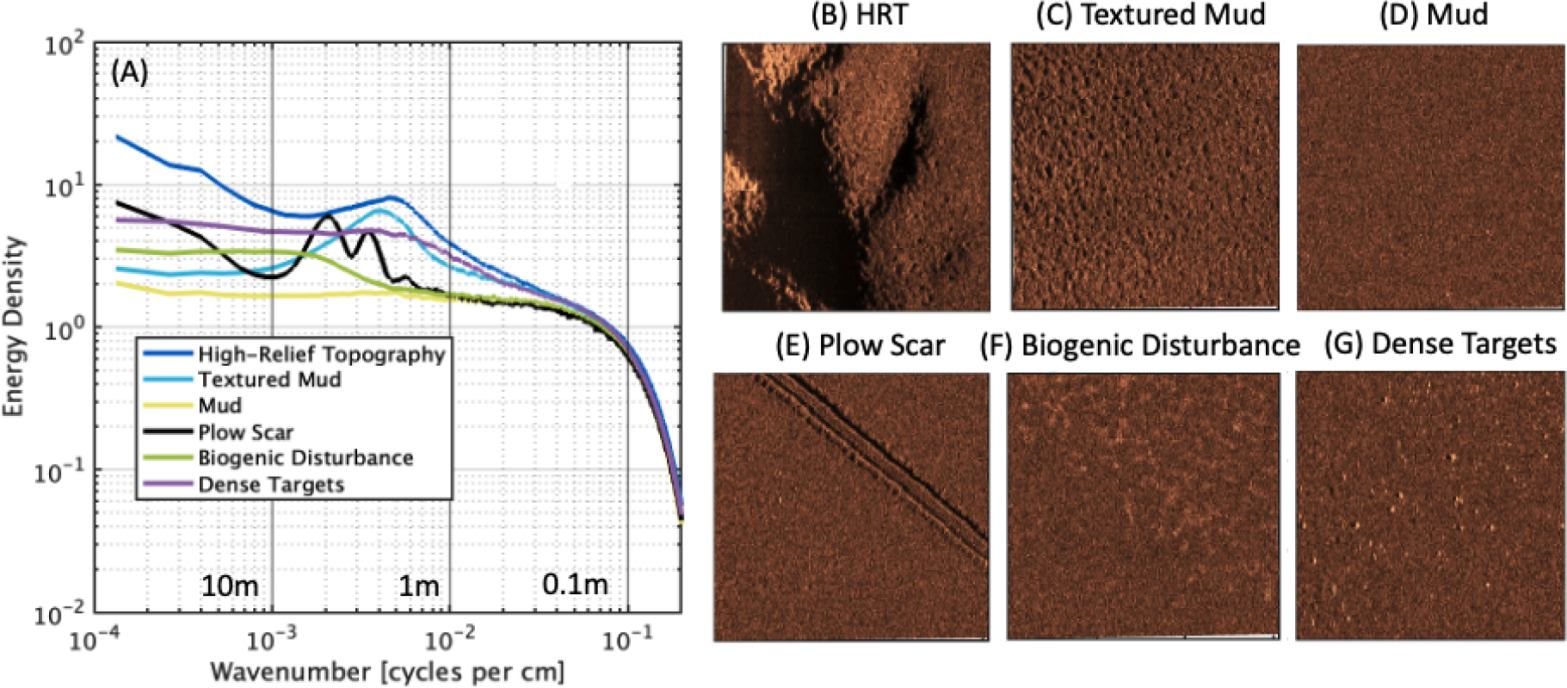
(A) Wavenumber spectra of classes used for seabed characterization. (B-G) 60 m × 60 m square boxes of normalized acoustic backscatter showing the corresponding seabed class to spectra in (A).

After file preprocessing, seabed variability is characterized by wavenumber spectral analysis and using statistical moments of distributions of acoustic intensity. To study seafloor variability that may have discrete spatial scales, a wavenumber spectrum is computed in the range direction for each preprocessed port and starboard ping. Averaging together a number (380–2000) of spectra reveals a set of unique spectral shapes that we associate with features of the seafloor (Figure 4). All spectra roll off at 0.1 m in the range direction representing the spatial noise floor or smallest detectable object in the data set.

Examples of acoustic images which correspond to the wavenumber spectra (Figure 4A) are shown in 60 m × 60 m boxes (Figure 4B-G). The high-relief topography (HRT) class (Figure 4B) is characterized by a spectrum that decreases with an increasing wavenumber and has high energy in spatial scales greater than 10 m with a secondary peak at 2.1 m. The textured mud class (Figure 4C) has a flat spectral shape with a broadband peak around 2.5 m. The bandwidth of the peak ranges from 1 to 6 m. The pattern of organized structures with wavenumbers of 1–6 m in both the textured mud and HRT classes suggests that the textured mud persists on larger geological features. The mud class (Figure 4D) is characterized by low variance and a flat wavenumber spectrum. The plow scar class (Figure 4E) has a distinctive bimodal structure associated with the trenching method with spectral peaks at 2.8 and 4.8 m. The fifth class (Figure 4F) has an increase in variance at scales of 5.5 m and longer but has low relief in the imagery (no shadows). We associate this pattern with a putative biogenic disturbance associated with bioturbation. The sixth class, dense targets (Figure 4G), shows small bright objects distributed throughout the image. Some of the objects have shadows, and the wavenumber spectrum shows increased variance at scales greater than 1 m.

To classify regions of the survey by the 6 spectral classes shown in Figure 4A, a machine learning technique is used. Feature representations of the wavenumber spectra are created by transforming the spectra with a Random Convolutional Kernel Transform (ROCKET) transformer.^39^ ROCKET utilizes random convolutional kernels with varying length, dilation, and padding to extract relevant information from the spectra (i.e., maximum value and proportion of the input that matches a given pattern). Prior to using the ROCKET transformer, wavenumber spectra were computed on every port and starboard side-scan ping and averaged over 25 along-track pings, corresponding to 12.5 m which was used for the seabed statistics below. The wavenumber spectra were also smoothed using a 5 band running average and cutoff at 1 m to remove roll-off associated with the noise floor before being transformed. Features extracted from ROCKET were input into a ridge regression classifier from scikit-learn.^40^ The ridge regression classifier converts the targets into multivariable regressions where individual ridge regression models are trained to distinguish one-vs-rest for each class. The predicted class is the output with the highest value. The ROCKET transformer was trained with 1000 random kernels using the 6 target classes (shown in Figure 4A). Features extracted from the ROCKET transformer are then used to train a ridge classifier to distinguish between the different classes. The ridge classifier uses a regularization strength of 1, and the solver was automatically chosen in scikit-learn. Both models are trained with the classes and data shown in Figure 4A.

In addition to spectral and machine learning seabed classification techniques, we compute statistics in 12.5 m × 12.5 m square boxes with 50% overlap. The resulting seabed maps are not overly sensitive to the choice of 12.5 m, but the choice of smaller boxes allows the investigation of patterns in the background on spatial scales of tens of meters. Variance, skewness, and entropy are computed over each box on the distribution of normalized acoustic intensities (see the Supporting Information for details). A 12.5 m × 12.5 m box has more than 23,000 acoustic values based on the along-track and cross-track resolution of the side-scan.

**Figure 5:**
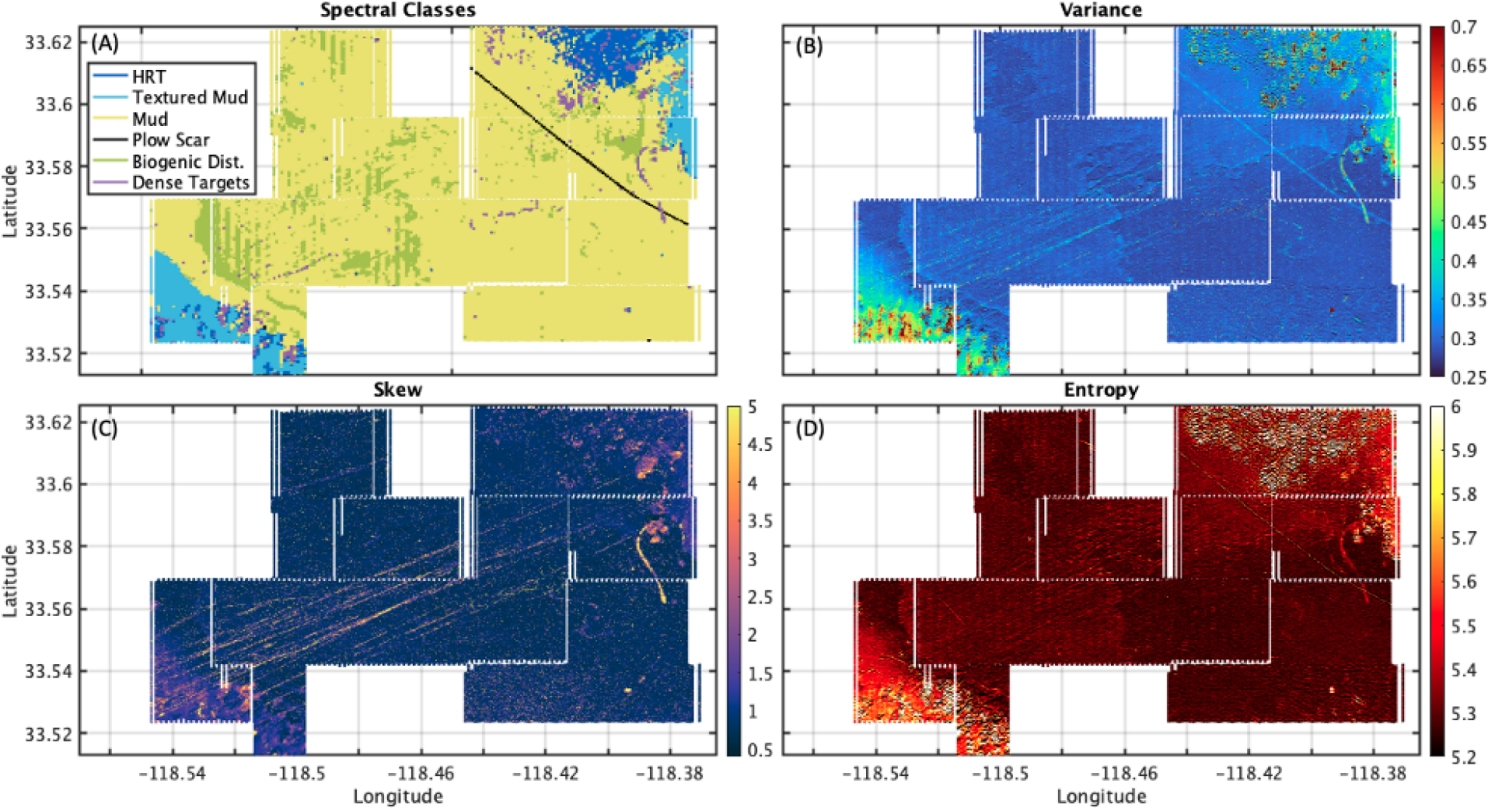
(A) Seabed map showing spectral classification using a supervised machine learning technique. The colors represent the 6 wavenumber spectral classes shown in Figure 4A. Statistical metrics, (B) variance, (C) skewness, and (D) entropy of distributions of acoustic intensity are computed in 12.5 m × 12.5 m boxes over the full survey. The metrics show patterns of targets and bottom type.

Results of wavenumber spectral (A) and statistical (B-D) classification are shown in Figure 5. The skill of the classifier is demonstrated by several key areas: 1) high-relief topography and textured mud seen in the NE and SW corners of the surveyed areas, 2) the plow scar is detected as an individual class and follows the known trace shown in Figure 5B, and 3) locations of the biogenic disturbances match large scale features in Figure 5B.

The statistical maps (Figure 5B-D) reveal large-scale patterns in seafloor variability. The highest values of variance are found in the regions of the survey on the sidewalls of the basin where the unconsolidated sediment cover is thin and bedrock exposures or rock fragments are present (Figure 5B). Lower variance values are found in the center of the survey where the seafloor is underlain by unconsolidated fine sediment. The areas with high entropy values correspond to areas with relatively rough bottom texture, whereas the flat bottom (basin floor) that characterizes most of the survey region has lower values. The northeast and southwest corners of the surveys, where the bathymetry begins to slope on the sidewalls of the basin, are characterized by high variance, high entropy, and intermittent regions of high skewness, variability that is associated with thin sedimentary cover over differentially eroded bedrock (Figure 5B-D). In the center of the survey, a distinct linear feature, oriented from northwest to southeast, is evident in the variance results (Figure 5B). This is a communications cable that runs from Santa Monica to San Diego, CA, and the resultant seabed disturbance from the trenching process remains visible. In the skewness map (Figure 5C), linear features with high values are oriented from northeast to southwest and extend throughout the survey bounds. A curved feature with high skewness is prominent on the east side of the survey, near the eastern basin sidewall slopes. Finally, a large-scale pattern in the deep portion of the basin, most prominently visible in the variance, lacks shadows (low skewness) indicating low relief differences. Further information on statistics as a function of seabed class are given in the Supporting Information. The resulting maps provide seafloor classification which is important for target detection and further studies investigating the biological and chemical composition of sediments in the region.

### 3.2 Target Detection and Classification

Objects within preprocessed side-scan images are identified using an acoustic anomaly detection technique (details in the Supporting Information). To design a classifier, debris targets identified by ROV imagery, observed in 2011/2013^14^ and 2021 surveys, are used for intensity and size criteria. Archival documentation of the containerized waste disposal practices is limited, so a number of container shapes and sizes are considered and listed in Table 2. The containers listed include dimensions of a 110-gallon drum and a long cylinder which were detected in the previous survey^14^ as well as the more common 55-gallon drum which may have been used for disposal of petroleum or other chemical waste.

**Table 2:** Target Descriptions

Target	Length	Width
55 gal. drum	0.86 m	0.58 m
110 gal. drum	1.08 m	0.77 cm
long cylinder	1.70 m	0.30 m

**Figure 6:**
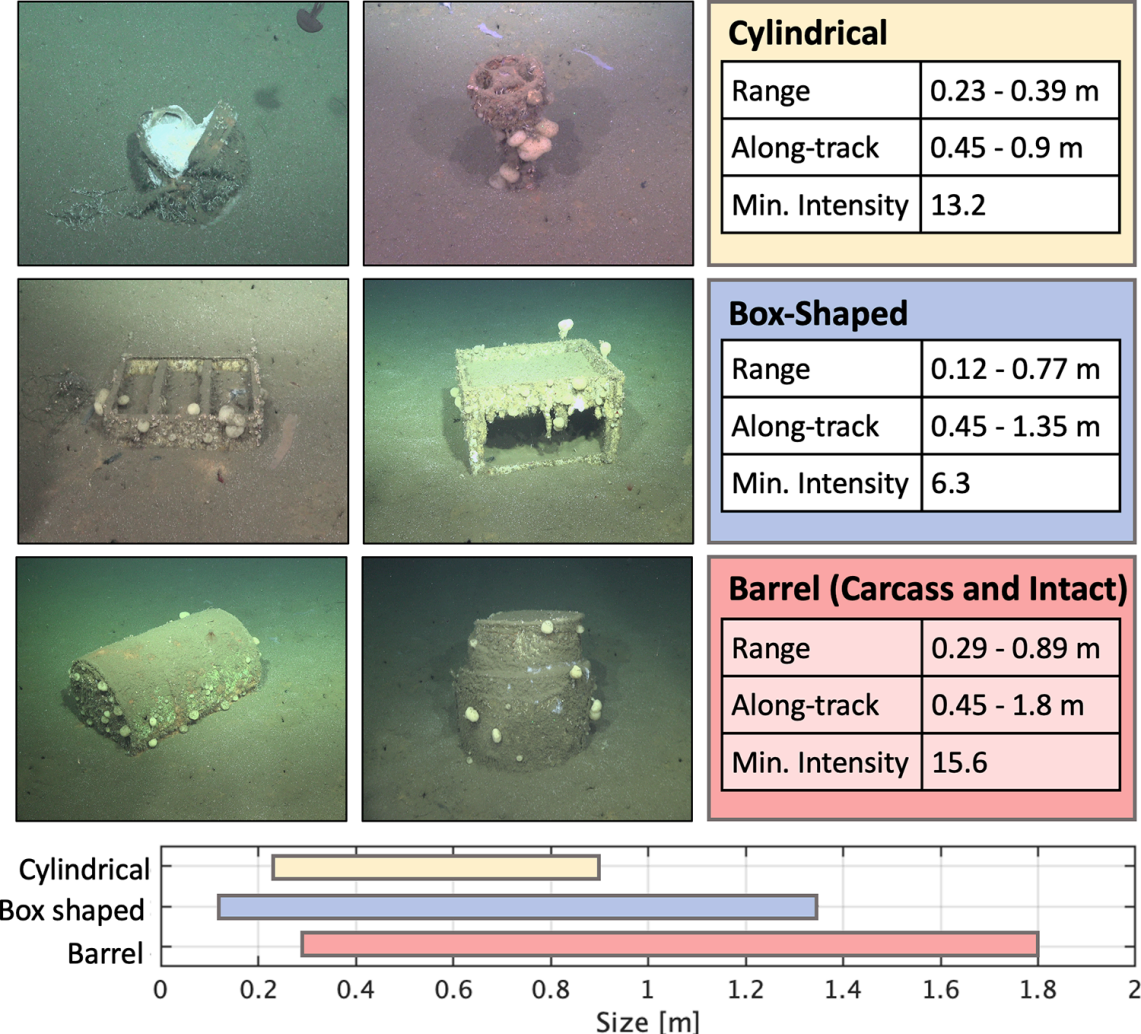
Across-track (range) size, along-track size, and minimum intensity criteria for different debris targets imaged with ROV SuBastian.

There were 60 barrels detected by ROV imagery in previous surveys within the dump site (Figure 1A).^14^ The survey spanned the previously mapped barrel locations resulting in a total of 121 detections of 53 unique barrels. Notably the same barrel will have different size and intensity characteristics from varying look angles dependent on the sonar’s grazing angle relative to the orientation and range of the object. In addition to barrel sized objects, a number of smaller seafloor debris targets were imaged by the 2021 ROV SuBastian survey. Two classes, a cylindrical form factor (tall, narrow target) and a box-shaped form factor, are shown with size and intensity characteristics relative to the barrels and are indistinguishable by dimension due to the resolution of the sonar. We find that both classes overlap with barrels in size but statistically have slightly lower minimum acoustic intensities (Figure 6).

Using the imagery and navigational information from ROV SuBastian, targets are located within the wide-area side-scan imagery. Navigational offsets were on average 8 m or less when collocating targets between the ROV and AUV data sets. Size and intensity characteristics of the debris targets were used to develop a classifier for the wide area survey data. The minimum and maximum size and intensity values of the validation targets are used to subset the detects based on criteria given in Table 2.

**Figure 7:**
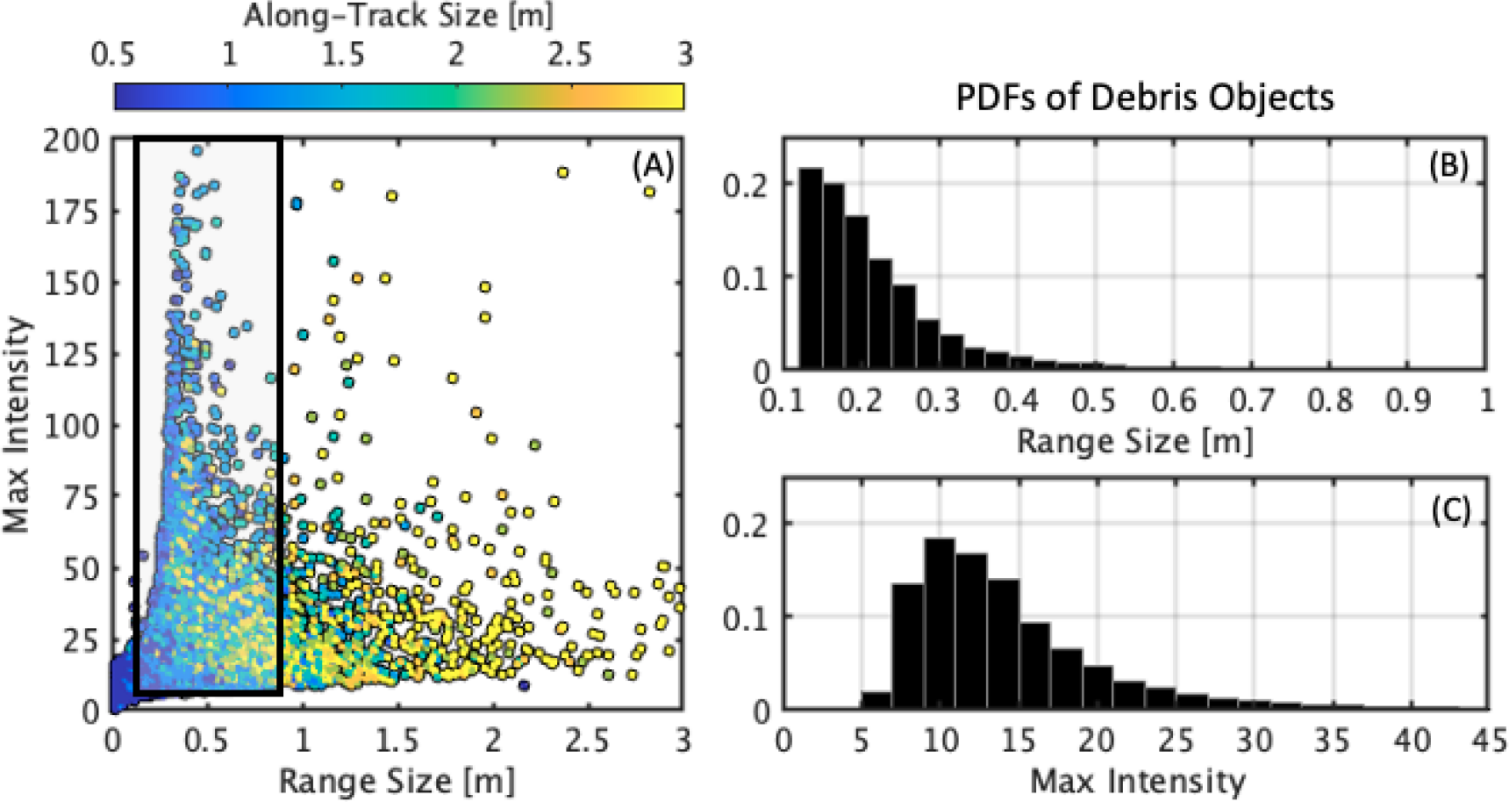
(A) Distribution of range pixels, along-track pixels (colors), and maximum intensity for all survey detects. The black shaded box represents the subset of targets classified as debris based on ROV footage. (B) and (C) show distributions of the debris-class targets for range and acoustic intensity.

Characteristics of the full set of targets, shown as a function of maximum normalized intensity, range size, and along-track size, are shown in Figure 7A. The distribution of all targets bifurcates into two branches, one with larger target sizes, shown by range and along-track sizes greater than 1 m and 2 m, respectively, and the other with smaller sizes but very high acoustic intensities. We interpret the former as larger objects associated with seafloor topography and the latter as small objects or electronic noise. The debris occupy the latter branch, with sizes ranging from 12 cm to 1.8 m (Figure 7A, shaded box). Probability Density Functions (PDFs) of the range size and acoustic intensity of the subset of objects that are classified as debris are shown in Figure 7B,C, respectively. The distributions show that the majority of debris-classified objects are small (10–20 cm) and may represent larger objects that are buried or degrading. The majority of debris targets has acoustic intensities between 8 and 15 suggesting that they have strong acoustic return over the background (scans were normalized to a mean acoustic intensity of 1). An advantage of our approach is that classification is a function of three metrics: range and along-track size dimensions and maximum acoustic intensity each of which can be adjusted to subset the full set of detections. For example, if there is interest in large, bright objects, our method allows for a map of the set of objects that satisfy user-specified size and intensity criteria.

### 3.3 Target Distributions and Maps

**Figure 8:**
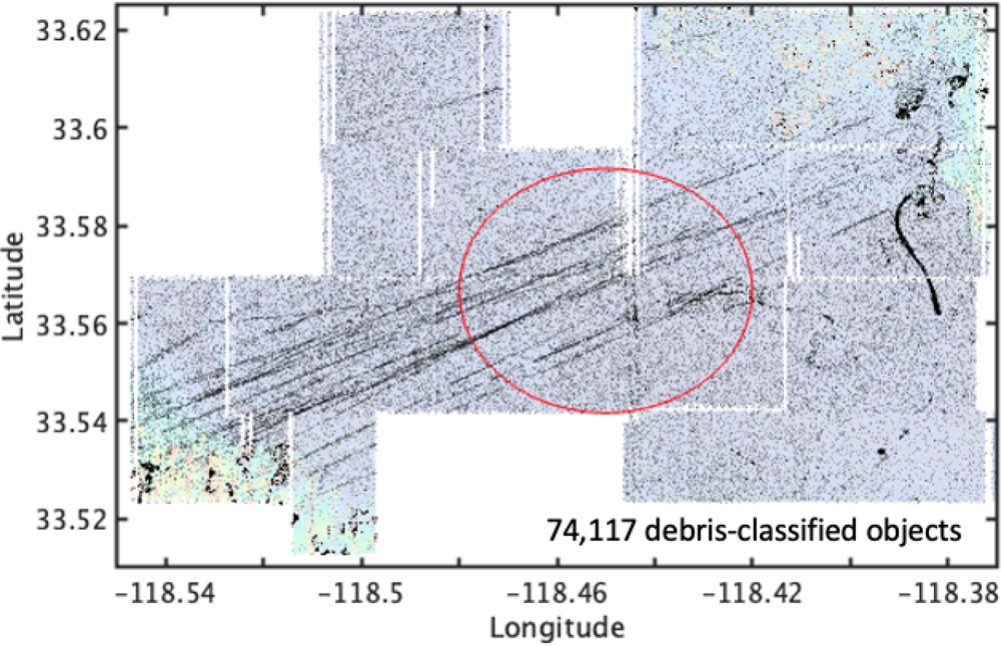
Subset of target detections that meet debris criteria as listed in Table 2. The background colors are the variance map from Figure 5B shown transparently. The red circle is dump site #2.

Maps of the debris objects based on the classifiers in Figure 6 are shown in Figure 8. The total number of targets detected in the data set, prior to mosaicing and large object removal, was over 140,000. This number is sensitive to the value of acoustic intensity threshold, but we have intentionally chosen a high value to focus on target anomalies that are significantly higher than the background. After removing targets in regions of high uncertainty due to bottom-type complexity (more details in the Supporting Information), 74,117 targets meet the debris criteria. Patterns in the target maps show linear features that span the full survey width from the northeast to southwest corner of the domain, exceeding the bounds of dump site #2 (Figure 8). The curved feature that is prominent in the seabed on the east side of the survey, between latitudes of 33.56° N and 33.59° N, appears to be densely populated with debris-classified targets. Another notable finding is that objects are found throughout the wide-area survey footprint indicating that the San Pedro Basin hosts a significant amount of debris on the seafloor.

### 3.4 Environmental and Societal Impacts

This study has focused on the development of techniques for mapping large areas of the seafloor with acoustic imaging from side-scan sonars on AUVs. For wide-area surveys, lower frequency systems are often used allowing swath widths of up to several hundred meters to be mapped on each pass. The resulting images have centimeter scale resolution in the cross-track of the vehicle and 10 s of centimeter resolution in the along track. This trade-off between the survey rate and resolution is an important consideration for seabed characterization. While higher frequency sensors provide higher resolution imaging and classification, the cost and time to cover wide areas increase significantly. In 2021, two AUVs mapped 150 km^2^ of the San Pedro Basin, CA, focusing on one of two known contaminant dump sites in the region. Previous work in the area had identified and imaged barrels, and sediment sampling in the region had found evidence of the chemical DDT and its breakdown products, a hazard to humans and marine ecosystems. Survey analytics included georeferencing, equalizing, and mosaicing raw side-scan imagery, characterizing the seafloor using statistical, spectral, and machine learning classification techniques, and identifying and classifying targets using acoustic thresholding and ground truth from previous surveys. The resulting map shows thousands of target objects distributed over the full survey domain. Linear patterns within the target maps suggest that ships leaving the Port of Long Beach, CA transited south approximately 4 km and then navigated on a fixed heading of approximately 240 degrees while dumping materials for several kilometers. Our wide area survey strategy was able to efficiently map linear features over 15 km, the scale of the San Pedro Basin.

Uncertainty remains as to the nature of the detected debris targets due in part to the diversity of California- and EPA-regulated industrial dumping, nonregulated munitions dumping,^6,41^ the resolution of our acoustic imaging sensors, and uncertainty if waste was containerized when dumped. Previous State and federal government reports^5,42,43^ outlined a process of large-scale disposal of DDT acid waste sludge in the San Pedro Basin containerized in 55-gallon barrels. According to some estimates, as many as 2,000 physical barrels per month were dumped in the San Pedro Basin from 1947 to 1961.^5^ While containerized dumping was not referenced in earlier peer-reviewed articles about the chemistry of the dump site,^12,13^ the recent reporting on the discovery of 60 barrels^14^ has led to a perception of widespread containerized dumping of DDT acid waste finding its way into the scientific literature^11^ and public media.^44−46^ However, recent research by the EPA^47,48^ that examined the sworn depositions of former Montrose chemical employees for evidence of disposal methods, suggests a practice by which byproduct DDT acid waste was in fact disposed of through bulk dumping instead of containerized barrels. Further efforts are required to understand the diversity and source of the objects (Figure 8) that have been mapped in this study.

Characterization of the seafloor can be used for object detection, e.g. clutter analysis, and for assessing bottom-type, sediment mobility, and the presence of biogenic activity. Carbonates associated with known methane seeps were found in the southwest corner of the survey; cold seeps are often associated with authigenic carbonates and are known for enhanced biological activity.^49^ Future surveys may leverage these findings for additional cold seep detection. Many of the barrels previously imaged by ROVs were surrounded by a hard alkaline precipitate (likely brucite – MgOH_2_) that probably formed when leaked contents interacted with sediment (K. Mizell, Pers. comm). This extended up to a meter from the barrel and was often buried 4–6 cm beneath the surface. This feature could have contributed to the acoustic signature and created a slightly expanded target dimension. Similarly, severe corrosion that changes the shape or integrity of barrels will change its acoustic signature. Previous ROV surveys^14^ revealed significant barrel degradation, with some barrels barely recognizable from their original shape. Waste disposal barrels (55 or 42 gallon drums) are fabricated from carbon steel sheet metal and have a finite life when submerged in seawater that depends on the amount of oxygen that supports steel oxidation. For the depths and oxygen levels at this site, we estimate a corrosion rate of 0.05 mm/year.^50^ Based on the Department of Transportation (DOT) regulations for the gauge of steel used in waste containers, we estimate the drums to have a useful containment life of 18–25 years. Since industrial dumping spanned four decades and ceased in the 1970s, the condition of observed waste containers from this era is expected to be compromised and will vary significantly depending on their age.

The presence of the large numbers of debris objects throughout the survey calls for additional characterization of the seabed objects and biological and chemical studies in the region. Physical oceanographic studies of bottom boundary layer dynamics, eddy transport, and basin exchange mechanisms are required to better understand the mobility of the contaminated sediments. The survey capabilities of AUVs provide an efficient mapping capability which will guide future studies that address the pathways for contaminant transport from deep ocean dump sites to the regional ecosystem and human health.

## Supporting Information Available

Additional data processing details, seafloor characterization statistics, and object detection methods (PDF)
