# Supplementary material for: Wide-Area Debris Field and Seabed Characterization of a Deep Ocean Dump Site Surveyed by Autonomous Underwater Vehicles
Source: Environ Sci Technol. 2023 Jun 15;57(46):18162–71. doi: 10.1021/acs.est.3c01256 (PMC10666539; doi:10.1021/acs.est.3c01256)
Supplement: Supplementary file 1 — es3c01256_si_001.pdf [file es3c01256_si_001.pdf]

Wide-area debris field and seabed characterization of a deep ocean dump site surveyed by autonomous underwater vehicles

Sophia T. Merrifield,[*,†] Sean Celona,[†] Ryan A. McCarthy,[†] Andrew Pietruszka,[†] Heidi Batchelor,[†] Robert Hess,[†] Andrew Nager,[†] Raymond Young,[†] Kurt Sadorf,[†] Lisa A. Levin,[†] David L. Valentine,[‡] James E. Conrad,[¶] and Eric J. Terrill,[†]

†*Scripps Institution of Oceanography, La Jolla, CA, 92037, United States*

‡*University of California, Santa Barbara, Santa Barbara, CA, 93106, United States*

¶*U.S. Geological Survey, Pacific Coastal and Marine Science Center, Santa Cruz, CA, 95060, United States*

E-mail: smerrifield@ucsd.edu

Phone: +1 858 246 2585

Supplementary Information

1 Sidescan Sonar Pre-processing

AUVs with integrated sidescan sonars have port and starboard transducer arrays which transmit narrow acoustic beams that illuminate the seafloor. Range-gated processing of the backscattered signal, combined with subsequent pings from the moving platform, are used to create images of the seabed. To account for transmission loss due to spreading and absorption, time-varying gain (TVG) is generally applied to individual ping data. Additional gain adjustments can be applied to improve image contrast; the challenge in wide area surveys is to develop target detection metrics that account for the very large dynamic range that occurs from local variations in bottom type (bright or weak signal return) but also allows for debris target detection across the survey domain.

During the April 2021 survey, over 160GB of raw sidescan data were collected onboard the AUVs. Several pre-processing steps are performed on raw files prior to developing automatic target recognition (ATR) algorithms. Vehicle heading rate is used to identify and remove pings that occur during vehicle turns which lead to distorted imagery. Portions of the survey where the vehicle exceeded a turn rate of 2.5°/sec are removed. Next, slant range, pitch, and roll corrections are performed to eliminate image artifacts that arise from vehicle motion. The Bluefin and Remus surveyed at 15 m and 20 m altitude off the seafloor, respectively.

To equalize pixel intensity as a function of range, pixel intensities are normalized by the average of 60 bands in the range direction and 200 bands in the along-track direction with 50% overlap in both directions. This serves to remove any pixel intensity range dependence while also adapting to local features within the image (Figure 2). The sequences of pings form images that are georeferenced to a longitude and latitude grid using the along track position information, vehicle speed, and cross-track range values.

Finally, the survey was run with 200% overlap such that the same area was imaged

twice, at different cross track ranges from the transducer array. Cross-track variability in backscatter intensity is due to range- and angular-dependent factors including seafloor bottom-type and the sonar beam pattern.[1] In this study, objects of interest are small (a few pixels or < 1.5 m) and the majority of bottom-types are sediment-covered and flat such that techniques to register and fuse[2] overlapping segments can lead to double counting of targets. For this reason, we choose to use sections of each scan at ranges 25-75% of the total swath width. For the Bluefin with 150 m swath width, this retains portions of the image at 37.5-112.5 m range and for the REMUS with 200 m swath width, ranges of 50-150 m from the transducers. This choice excludes side lobe interference at close ranges and eliminates geometric distortion at the furthest ranges of the image. The resulting images are stitched together and have no overlap.

2 Statistics

Statistical metrics provide information on large-scale patterns in the wide-area sidescan survey. The following information describes the definitions of variance, skewness, and entropy and how each relates to sidescan data. The statistics are computed using the pixel intensities, x, contained within each of the defined boxes.

Variance (V) is defined as:

$$V = \frac{1}{N-1}\sum_{i=1}^{N}|x_i - \nu|^2, \tag{1}$$

where ν is the mean of A, and N represents the number of values in the distribution. High variance may be associated with the presence of targets or geological features. Skewness (S) is defined as:

$$S = \frac{E(x-\nu)^3}{\sigma^3}, \tag{2}$$

where ν is the mean of x, σ is the standard deviation of x and $E(t)$ represents the expected

value of the quantity t. Skewness is the statistical measurement of the asymmetry of a probability distribution. Areas of high skewness are associated with targets or geology which produce shadows shifting the intensity distribution towards lower values. Entropy (H) is defined as:

$$H = -\sum_{i=0}^{N-1} p(z_i) log_2 p(z_i), \tag{3}$$

where p contains the normalized histogram counts of the data, x. H provides a statistical metric for texture or randomness within a box.

While the range-dependent mean of each file has been equalized during pre-processing, the range-dependence of other statistical metrics may still be present. To eliminate range-dependence, the median of the data in each 12.5 m x 12.5 m box is corrected relative to the value nearest to the nadir. This process has a minor impact on the statistical values, but reduces range-dependence in the full survey map visualization.

3 Seabed Characterization

After determining the spectral classification of the full seafloor survey, we consider the distributions of the statistical metrics by class. Normalized distributions of variance, skewness, and entropy are shown as a function of seafloor class (Figure 1A-R). Notably, the HRT and mud classes are characterized by broad distributions of variance (Figure 1A,D) and entropy (Figure 1C,F), and higher values of skewness (Figure 1B,E) relative to the distribution of all survey values. Mud, which accounts for the greatest area of the seafloor classification, is characterized by low variance, low skewness, and a narrow distribution of entropy (Figure 1G-I). The plow scar and biogenic disturbance have slightly elevated values of variance and skew, with an additional tail of high entropy values for the plow scar (Figure 1J-O). Finally, dense targets distributions have large tails in variance and skewness, but the values of entropy are lower and more narrowly distributed than for the textured mud class (Figure 1P-R). This becomes important for distinguishing background clutter associated with high-

relief topography and textured mud versus small bright targets associated with debris on the seafloor.

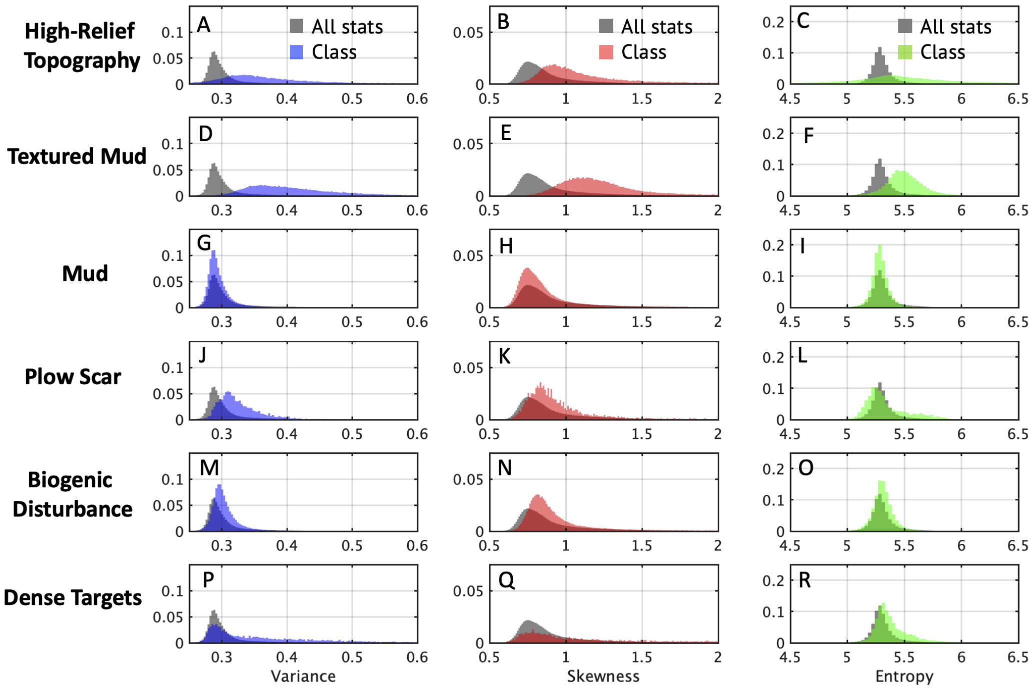

Figure 1: Statistical metrics of variance (blue), skewness (red), and entropy (green) are grouped into normalized probability distributions by spectral class. Each class distribution is shown relative to the full survey distribution (grey) for reference.

4 Target Detection

Prior to anomaly detection, a convolution filter is applied to the range-dependent intensities to reduce noise in the image. The ratio of the along-track to range pixel dimensions is approximately 27:1 for the Bluefin and 30:1 for the REMUS, so 27-point and 30-point Blackman windows are chosen and applied only in the range-direction, respectively. The Blackman window, a generalized cosine window, is defined as:

$$w(n) = 0.42 - 0.5 \cos \frac{2\pi n}{N-1} + 0.08 \cos \frac{4\pi n}{N-1}, \tag{4}$$

where N is the window length and n is defined between $[0, \frac{(N+1)}{2} - 1]$.

Each sidescan file is binarized using an intensity threshold of 4 and targets are counted as independent, connected components in the binary image (Figure 2). The threshold value is chosen to represent 3-5 standard deviations from the mean for most of the survey. Size and intensity characteristics of each target are stored including the number of cross-track pixels, number of along-track pixels, total number of pixels, maximum intensity from the pre-processed file (pre-filtering), and longitude and latitude position of the targets. Along and across track pixels are converted to size using the pixel resolutions of the sidescan sonar listed in Table 1.

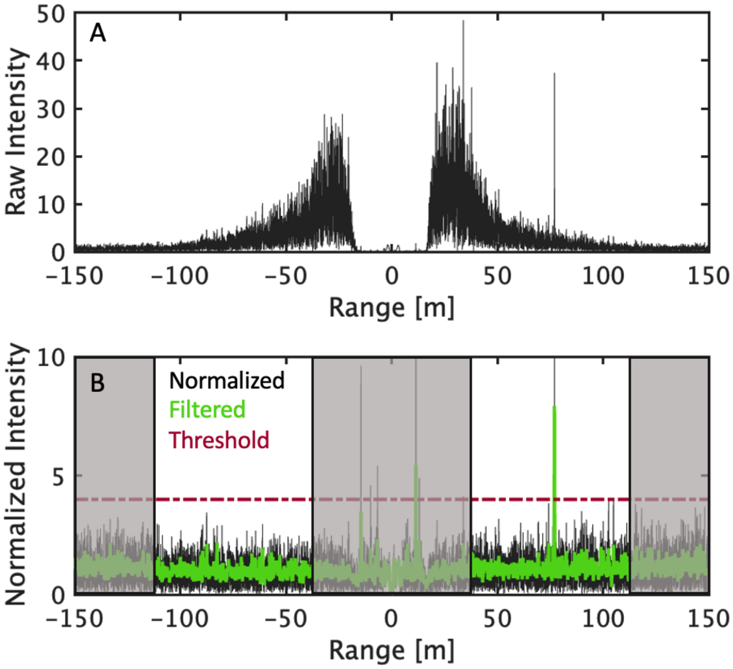

Figure 2: (A) Raw intensity as a function of range for one sidescan ping to port and starboard. (B) Pre-processed intensity includes normalization as a function of range, choice of the main lobe section (white), convolution filtering (green), and an intensity threshold (red) to identify targets.

4.1 Target Distributions and Maps

Detection of small targets in a wide area survey is more difficult in the presence of large objects, such as wrecks, and geological features. The sidewalls of the San Pedro Basin, the southwest and northeast portions of the wide area survey, have the most complex seafloor with small-scale variability in acoustic return. Areas with large rocks or a shipwreck often have dense, discontinuous, acoustic returns which present as small bright targets, similar to the targets of interest in this study. To account for seabed complexity, where it will require higher resolution acoustic and optical imagery to resolutely distinguish between targets and the background, we design a mask to remove targets in regions of known uncertainty. We use an entropy and class-based metric, computed in 12.5 m x 12.5 m seabed boxes, corresponding to the maps in the previous section. We select HRT and textured mud seabed classes and further subset using an entropy criteria to isolate the tails, values less than 5.15 and greater than 5.6 are removed from the target detections.

References

(1) Capus, C.; Banks, A.; Coiras, E.; Ruiz, I. T.; Smith, C.; Petillot, Y. Data correction for visualisation and classification of sidescan SONAR imagery. *IET radar, sonar & navigation* **2008**, *2*, 155–169, Publisher: IET.

(2) Reed, S.; Ruiz, I. T.; Capus, C.; Petillot, Y. The fusion of large scale classified side-scan sonar image mosaics. *IEEE transactions on image processing* **2006**, *15*, 2049–2060, Publisher: IEEE.
